# Supplementary figures and images for: Nuclear Fragility in Radiation-Induced Senescence: Blebs and Tubes Visualized by 3D Electron Microscopy
Source: Cells. 2022 Jan 13;11(2):273. doi: 10.3390/cells11020273 (PMC8774169; doi:10.3390/cells11020273)

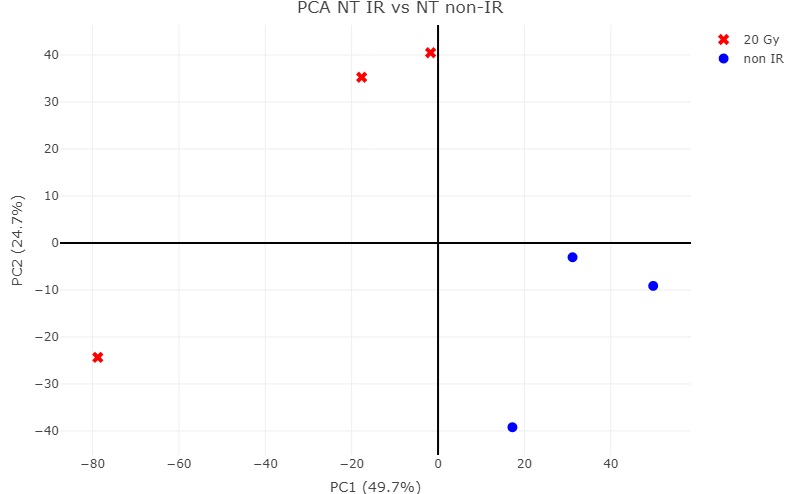

Supplement: Supplementary file 1 [file cells-11-00273-s001.zip › Supplementary_data/Supplementary Figure 1.jpg]

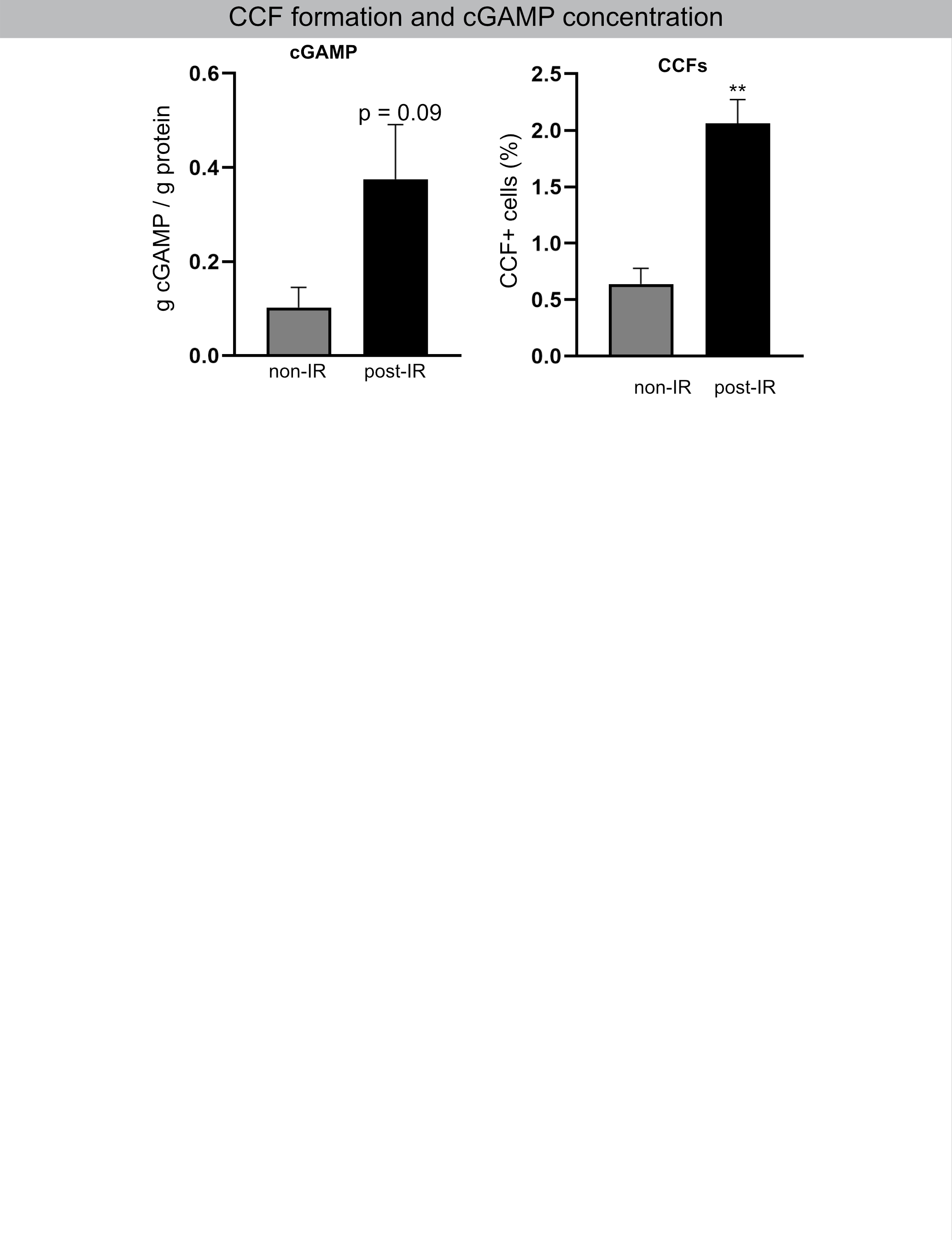

Supplement: Supplementary file 1 [file cells-11-00273-s001.zip › Supplementary_data/Supplementary Figure 2.tiff]

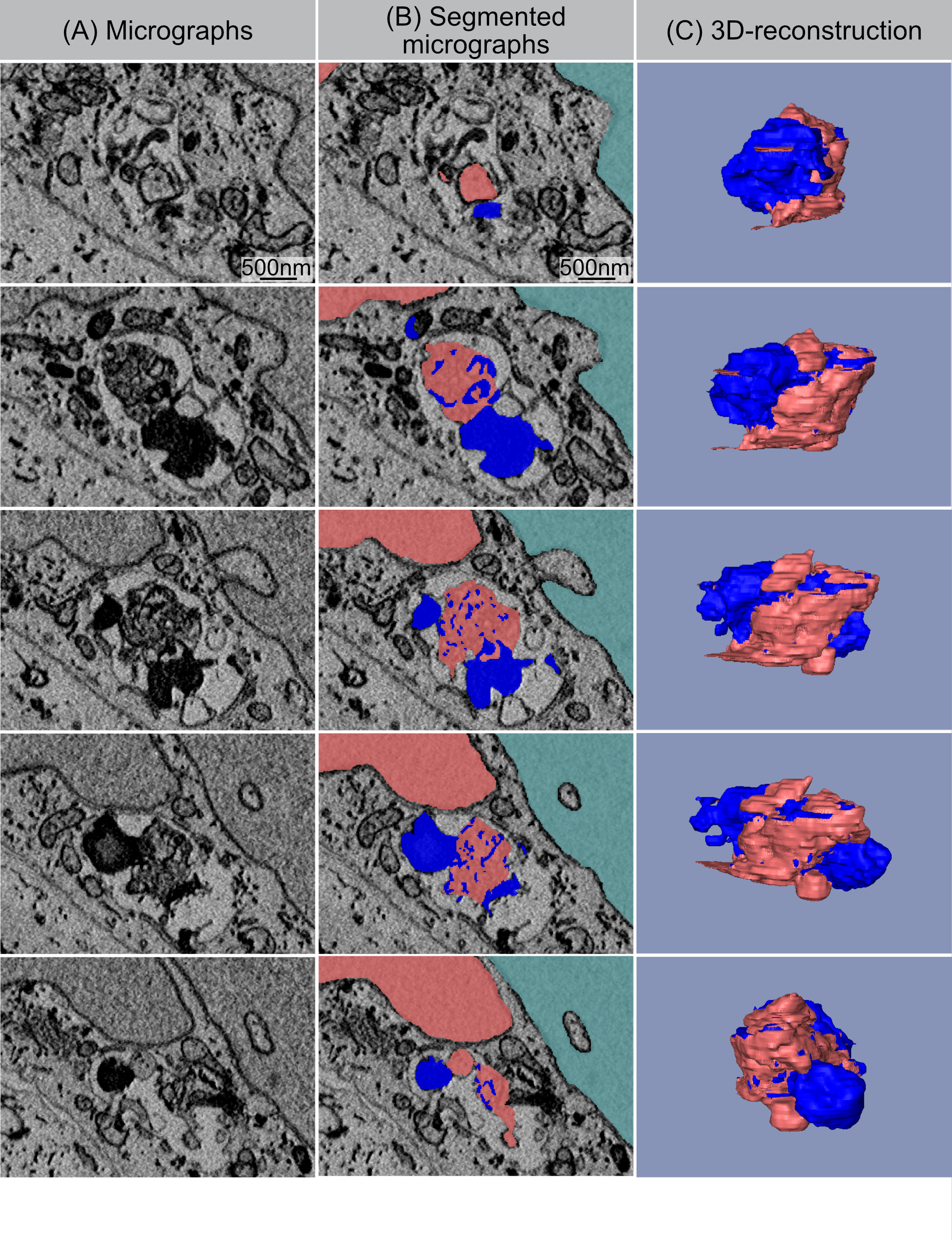

Supplement: Supplementary file 1 [file cells-11-00273-s001.zip › Supplementary_data/Supplementary Figure 3.tiff]
